# Health-economic evaluation of orthogeriatric co-management for patients with forearm or humerus fractures: an analysis of insurance claims data from Germany

**DOI:** 10.1186/s12913-024-11297-1

**Published:** 2024-07-16

**Authors:** Espen Henken, Hans-Helmut König, Clemens Becker, Gisela Büchele, Thomas Friess, Andrea Jaensch, Kilian Rapp, Dietrich Rothenbacher, Claudia Konnopka

**Affiliations:** 1 Department of Health Economics and Health Services Research, University Medical Center Hamburg-Eppendorf, Martinistr. 52, 20246 Hamburg, Germany; 2 Department of Clinical Gerontology, Robert-Bosch-Hospital, Stuttgart, Germany; 3 Institute of Epidemiology and Medical Biometry, Ulm University, Ulm, Germany; 4 AUC - Akademie Der Unfallchirurgie GmbHAUC - Akademie der Unfallchirurgie GmbH, Munich, Germany

**Keywords:** Orthogeriatric co-management, Humerus fractures, Forearm fractures, Health-economic evaluation

## Abstract

Orthogeriatric co-management (OGCM) describes a collaboration of orthopedic surgeons and geriatricians for the treatment of fragility fractures in geriatric patients. While its cost-effectiveness for hip fractures has been widely investigated, research focusing on fractures of the upper extremities is lacking. Thus, we conducted a health economic evaluation of treatment in OGCM hospitals for forearm and humerus fractures.

In a retrospective cohort study with nationwide health insurance claims data, we selected the first inpatient stay due to a forearm or humerus fracture in 2014–2018 either treated in hospitals that were able to offer OGCM (OGCM group) or not (non-OGCM group) and applied a 1-year follow-up. We included 31,557 cases with forearm (63.1% OGCM group) and 39,093 cases with humerus fractures (63.9% OGCM group) and balanced relevant covariates using entropy balancing. We investigated costs in different health sectors, length of stay, and cost-effectiveness regarding total cost per life year or fracture-free life year gained.

In both fracture cohorts, initial hospital stay, inpatient stay, and total costs were higher in OGCM than in non-OGCM hospitals. For neither cohort nor effectiveness outcome, the probability that treatment in OGCM hospitals was cost-effective exceeded 95% for a willingness-to-pay of up to €150,000.

We did not find distinct benefits of treatment in OGCM hospitals. Assigning cases to study groups on hospital-level and using life years and fracture-free life years, which might not adequately reflect the manifold ways these fractures affect the patients’ health, as effectiveness outcomes, might have underestimated the effectiveness of treatment in OGCM hospitals.

**Supplementary Information:**

The online version contains supplementary material available at 10.1186/s12913-024-11297-1.

## Background

Fragility fractures constitute the clinical significance of osteoporosis [1]. They are associated with a substantial health and economic burden [2, 3], especially in aging societies as one of the most important risk factors for fragility fractures is higher age [2]. In Germany, the costs of fragility fractures made up about 3.7% of the healthcare spending in 2019, and due to demographic changes they are expected to increase further [4]. Adverse health outcomes of fragility fractures include pain [5], loss of functioning [6], institutionalization [7], and death [8]. The extent of health and economic consequences depend on the fracture location with hip and vertebral fractures being the most severe [9]. Therefore, hip fractures were the focus of most studies on fragility fractures [10] while other fractures’ impact on public health might be underestimated [11–13]. Two very common non-hip, non-vertebral fragility fractures are forearm and humerus fractures [2, 3]. Despite their lesser impact on mortality than hip or vertebral fractures [11, 14, 15], they pose a big public health burden [11, 15]. Forearm and humerus fractures were responsible for 33% of the costs of fragility fractures in 6 countries in the European Union [3]. Moreover, a cross-sectional analysis found that irrespective of the initial fracture location, the risk for a subsequent fracture was high [16].

A comprehensive treatment of fragility fractures must account for the patients’ frailty as this is closely related to the resulting health and economic consequences. In a recent study, patients with fragility fractures had 7–9 comorbidities [15]. To address this, holistic treatments such as orthogeriatric co-management (OGCM) have been developed [17–21]. OGCM in Germany involves a geriatrician-led multidisciplinary team of orthopedic surgeons, physiotherapists, occupational therapists, specially trained nurses, and social workers [22]. It includes a standardized geriatric assessment, regular team meetings, the development of a rehabilitation plan, and early mobilization [22] and is applied either jointly in an orthogeriatric unit or as a geriatric liaison service in an orthopedic ward with an early transfer to a geriatric ward. It can be reimbursed using the operation and procedure code OPS8-550 – complex early geriatric rehabilitation [22].

For hip fractures orthogeriatric care was associated with decreases in hospital and 1-year mortality, higher treatment rates for osteoporosis, decreased healthcare costs, and suggested to be cost-effective [23–25]. Lower costs might be explained by a shorter length of stay (LOS) in the orthogeriatric care groups [23, 24]. However, none of the German studies obtained a significant decrease in LOS [22, 26–30] while some found an increase [22, 26, 27]. Consequently, a German health-economic evaluation based on claims data found cost-effectiveness of OGCM only at €82,000 per life year gained [26]. Nevertheless, studies about OGCM for fractures of the upper extremities are rare. A German prospective cohort study compared multiple outcomes of patients with major (mostly lower extremities, hip, and pelvic fractures) and minor osteoporotic fractures (mostly upper extremities) treated with OGCM [31]. As they found similar results in both groups, they argued that minor fractures should also receive multidisciplinary care – a call already made by other researchers [32, 33]. As, in Germany, OGCM is already applied for forearm and humerus fractures, hospitals that already implemented OGCM, can be compared with those that have not yet implemented OGCM. Thus, this study compared costs and cost-effectiveness associated with treating forearm and humerus fractures in an OGCM or a non-OGCM hospital.

## Methods

We conducted a retrospective cohort study with complete health and long-term, nationwide insurance claims data from 2013–2019 from the WIdO (*Wissenschaftliches Institut der AOK*), the scientific institute of the *Allgemeine Ortskrankenkasse (AOK)*. The study did not follow a published health economic analysis plan. We selected inpatient forearm or humerus fracture cases in 2014–2018. We included stays with a discharge ICD-10 code of S52 (forearm fracture), S42 (humerus fracture), or M80 (osteoporosis with pathological fracture). Inpatient stays with M80 discharge diagnosis were only included if they were accompanied by a relevant admission diagnosis or a single relevant secondary diagnosis. We considered forearm (S52), humerus (S42), pelvic (S32.1, S32.3, S32.4, S32.5, S32.81, S32.83), vertebral (S12.0, S12.1, S12.2, S12.7, S.12.9, S22.0, S22.1, S32.0), or hip fractures (S72.0, S72.1) as secondary diagnoses. Thus, cases with M80 as discharge and forearm or humerus fractures as secondary diagnosis were only included if none of the other fractures were recorded as secondary diagnoses.

Identified cases were either treated in an OGCM hospital (OGCM group) or a non-OGCM hospital (non-OGCM group), based on a categorization provided by the WIdO that defined OGCM hospitals as those with at least 10 operation and procedure codes OPS8-550 within a year (independently of the type of fracture). Hospitals not categorized as OGCM hospital in one but in subsequent and preceding years were also defined as OGCM hospitals. OPS8-550 can only be coded if the treatment lasted for 7 (8–550.0), 14 (8–550.1), or 21 days (8–550.2) and the 14 days threshold is particularly relevant for a higher reimbursement rate [34]. We applied this hospital-level approach, considering that patients who did not survive or were not treated as long as these thresholds would be assigned to the non-OGCM group if we had used this OPS code for group assignment on patient level. We assume that patients treated in hospitals with the expertise to offer OGCM treatment will benefit from these structures irrespective of whether OPS8-550 was reimbursed. We used the year before admission to the first relevant inpatient stay (*index stay*) as baseline and the following year as follow-up period.

**Fig. 1:**
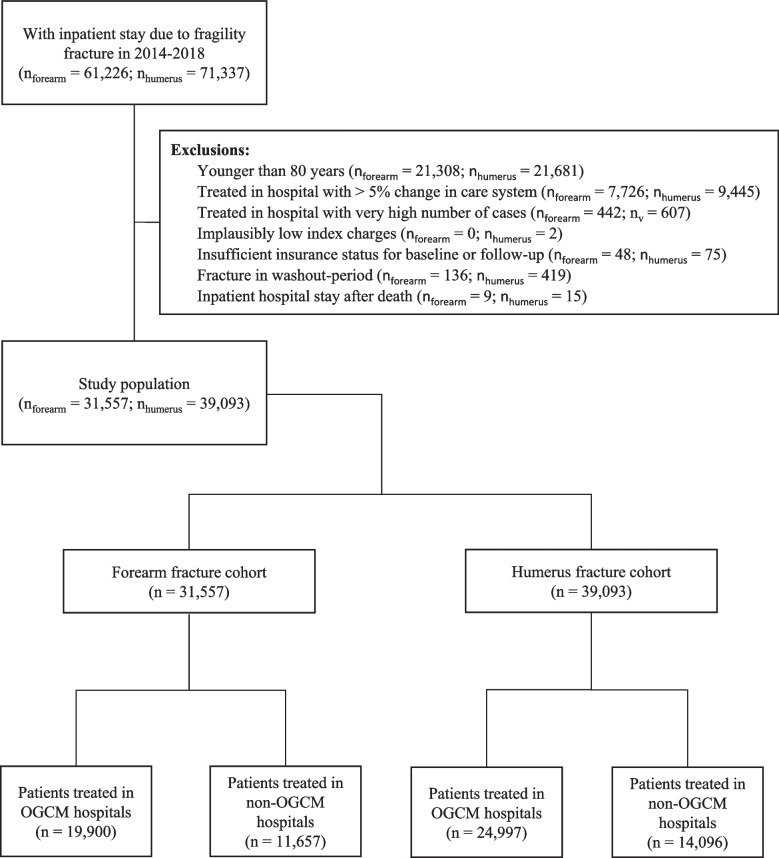
Flow-chart of the study populations

Figure 1 shows a flowchart of the sample selection: We only considered continuously insured patients (at least 90 days within a quarter and 360 days within a year) and excluded patients younger than 80 years at admission as OGCM was designed for a geriatric population. As it was unclear what part of the treatment was applied in which hospital when patients were transferred during their stay, we excluded all cases treated in hospitals that frequently (> 5% of the cases) transferred forearm or humerus fracture patients to hospitals with different OGCM status (OGCM hospitals transferring to non-OGCM hospitals and vice versa). To allow for an adequate balancing of hospital volume (the number of fracture cases per hospital during the study period used as an approximation for hospital size), we had to exclude all cases treated in a hospital with a particularly high number of fractures. In addition, we excluded *n* = 2 patients in the humerus fracture cohort whose index stay costs were implausibly low (€0.01). Moreover, we excluded all cases of patients not insured for the entire baseline or follow-up. We only used the first fracture case per person and fracture location. Then, we applied a washout period of 180 days before the selected fracture cases excluding all patients with an inpatient stay due to the same fracture location. Lastly, we excluded all patients with a hospital stay recorded after the estimated day of death.

### Outcomes

We analyzed the following cost sectors: inpatient hospital (including inpatient rehabilitation and the index stay), outpatient and outpatient hospital, medication, medical devices/medical appliances, long-term care, and total costs, the sum of all sectors. We also analyzed the index stay separately (including costs for consecutive stays with the same fragility fracture and inpatient rehabilitation costs of stays starting 4 weeks after hospital discharge). Besides from the index stay, which captures the costs of the initial fracture, we did not limit the scope of costs to those caused by the respective fracture as they are difficult to assign. We calculated long-term care costs indirectly because we received care data on a monthly basis without the associated costs (see Additional File 1, appendix A, p. 1). All costs stem from a payer perspective, were not discounted due to the follow-up of only one year, and are given in 2019 Euro, inflation-adjusted using the Gross Domestic Product price index [35]. We winsorized costs at the 99% percentile to limit the influence of extreme outliers. Furthermore, we analyzed in-hospital LOS, the LOS in a rehabilitation facility (considering the first rehabilitation stay 28 days after index stay discharge), and the total LOS – the sum of both.

We used *life years* – the proportion of the 1-year follow-up a person survived – as effectiveness outcome. As we only received information on patients’ deaths per monthly period, we used the last insured day of deceased patients to approximate the date of death. Additionally, we used the survived proportion of the follow-up without subsequent fragility fracture as effectiveness outcome (*fracture-free life years)*. We considered all in- or outpatient hospital treatments due to forearm, humerus, pelvic, vertebral, or hip fractures as subsequent fractures. We assumed diagnoses of the same fracture type as the initial fracture within 6 weeks after the index stay, retreatments rather than refractures, and did not consider them.

### Risk adjustment

To minimize the influence of selection bias and imbalanced covariate distributions due to a lack of randomization, we applied entropy balancing [36]. It weights the individuals in the non-OGCM group in such a way that the mean, variance, and skewness of covariates closely match those in the OGCM group. Entropy balancing has been shown to produce superior balance than similar methods such as weighting or matching with propensity scores [37]. We used the following covariates: Sex and age at the index date, 22 medication-based chronic health conditions during baseline [38], treatment year, months within each care level and months in a nursing home during baseline, costs from all health sectors during baseline, and hospital volume (at hospital level; see Additional File 1, appendix A, p.1). To achieve adequate balancing, we had to exclude a hospital with a very high hospital volume for both fracture types (see supplementary Fig. 1, Additional File 1, appendix B, p.2). Moreover, we excluded HIV, tuberculosis, and migraines from balancing as they occurred less than 50 times in each cohort to obtain sufficient overlap between groups.

### Statistical analyses

We used the entropy balancing weights for all analyses. We used weighted generalized linear models with gamma distribution to analyze the total and in-hospital LOS as well as total, inpatient, and index costs accounting for the typically right-skewed distributions. While these outcomes only contained positive non-zero values because an inpatient hospital stay due to a fragility fracture was a selection criterion, other cost sectors included a substantial proportion of zeros. To analyze outpatient and outpatient hospital, medication, and medical devices/medical appliances costs, we used weighted two-part models with logistic regressions in the first part to analyze whether costs occurred and generalized linear regressions with gamma distribution to analyze the amount of costs [39]. We also analyzed the LOS in a rehabilitation facility with this model. As neither the effect measures nor the long-term care costs showed a right-skew, we used weighted linear regressions here.

We calculated incremental cost-effectiveness ratios (ICER) as the ratio of the difference in weighted mean total costs between OGCM and non-OGCM and either the difference in weighted mean life years or fracture-free life years gained. To estimate the probability that treatment in an OGCM hospital was cost-effective at different willingness-to-pay (WTP) thresholds, we used the net-monetary regression approach [40, 41] with robust standard errors, and iterating the WTP from €0 to €150,000 by €1,000 steps. We displayed the results in cost-effectiveness acceptability curves (CEAC; [41]) considering the intervention to be cost-effective if a 95% probability was exceeded.

To account for potential clusters due to cases treated in the same hospitals, we conducted sensitivity analyses recalculating all analyses using a random intercept term for hospitals. As balancing for hospital volume led to high weights for patients in non-OGCM hospitals with a large volume, we calculated a sensitivity analysis without balancing for hospital volume. For sample description, we report rates of cases with an OPS code indicating OGCM treatment (OPS8-550) and rates of cases with an OPS code indicating a surgical procedure(see Additional file 1, appendix A, p. 1). We set α = 0.05 and used R (version 4.2.0), SAS software v9.4 (SAS Institute Inc, Cary, NC), and Stata 16 (StataCorp, College Station, TX) for all analyses.

## Results

We included 31,557 cases of patients with forearm (63.1% OGCM group) and 39,093 cases with humerus fractures (63.9% OGCM group). Means and standard deviations of covariates before and after entropy balancing are depicted in supplementary Tables 1 and 2 (Additional File 1, appendix C, p. 3–6.). An OPS8-550 was reimbursed in 8.9% of the OGCM and < 0.1% of the non-OGCM group in the forearm fracture and 22.6% and < 0.1% in the humerus fracture cohort. For 85.4% of the cases in the forearm fracture and 61.8% in the humerus fracture cohort an OPS code that indicated surgical treatment was recorded.

**Fig. 2:**
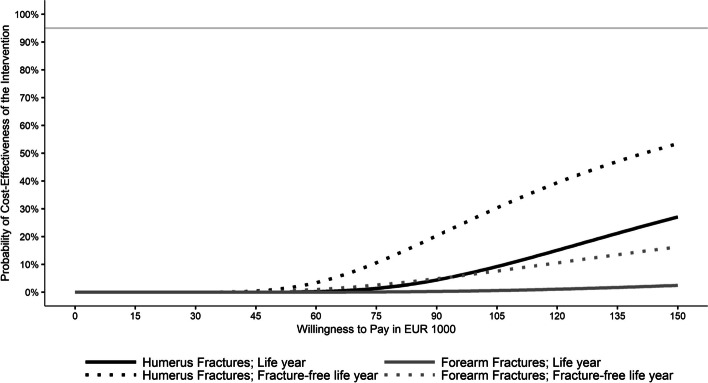
Cost-effectiveness acceptability curves for total costs per (fracture-free) life year gained

The results for the forearm fracture cohort are depicted in Table 1. We found significantly higher total costs in the OGCM than in the non-OGCM group and no differences concerning either effectiveness outcome. The OGCM group was dominated by the non-OGCM group concerning life years gained and the ICER was €867,129 per fracture-free life year gained. The CEAC in Fig. 2 shows that the probability for the intervention to be cost-effective did never exceed 95%.

**Table 1:** Costs and outcome estimates for forearm fractures

Outcome	OGCM group (*n* = 19,900)	Non-OGCM group (*n* = 11,657)	Difference	SE
Costs [€]				
Total^a^	16,855	16,074	780^*^	164
Inpatient^a^	8,326	7,560	765^*^	100
Thereof during index stay^a^	4,201	3,667	534^*^	29.71
Medication^b^	1,084	1,085	-0.7849	18.85
Outpatient^b^	997	1,013	-15.92	9.53
Outpatient hospital^b^	34.06	36.3	-2.24	1.87
Medical devices^b^	314	314	-0.1647	6.37
Long-term care^c^	5,849	5,855	-6.33	95.43
Length of stay [days]				
Total stay^a^	8.21	6.42	1.79^*^	0.1119
Thereof in hospital^a^	7.79	6.08	1.71^*^	0.0967
Thereof in rehabilitation facility^b^	0.4167	0.3432	0.0735	0.0427
Effectiveness				
Life year^c^	0.93	0.9313	-0.0014	0.0032
Fracture-free life year^c^	0.8696	0.8687	0.0009	0.0041
ICER				
€ per life year gained	Dominated^d^			
€ per fracture-free life year gained	867,129			

*OGCM* Orthogeriatric co-management, *OGCM* Orthogeriatric co-management, *SE* Robust standard error

*p < .001

^a^Estimated with a gamma regression

^b^Estimated with a two-part model with logistic and gamma parts

^c^Estimated using a linear regression

^d^OGCM was more costly and less effective than non-OGCM group

The results for the humerus fracture cohort are displayed in Table 2. We found significantly higher total costs, no differences regarding life years, and slightly higher fracture-free life years in the OGCM group than in the non-OGCM group. The ICER was €233,646 per life year and €141,113 per fracture-free life year gained. The CEAC in Fig. 2 shows that the probability for the intervention to be cost-effective never exceeded 95%.

**Table 2:** Costs and outcome estimates for humerus fractures

Outcome	OGCM group (*n* = 24,997)	Non-OGCM group (*n* = 14,096)	Difference	SE
Costs [€]				
Total^a^	21,994	20,569	1425^**^	221
Inpatient^a^	11,715	10,555	1160^**^	162
Thereof during index stay^a^	6,776	5,736	1041^**^	68.5
Medication^b^	1,197	1,159	38.3	21.18
Outpatient^b^	964	960	3.62	12.26
Outpatient hospital^b^	27.7	23.71	3.99^*^	1.8
Medical devices^b^	445	462	-17	9.6
Long-term care^c^	7,377	7,163	214	119
Length of stay [days]				
Total stay^a^	15.8	12.04	3.76^**^	0.1988
Thereof in hospital^a^	13.79	10.12	3.67^**^	0.1417
Thereof in rehabilitation facility^b^	2.01	1.91	0.098	0.1178
Effectiveness				
Life year^c^	0.8533	0.8472	0.0061	0.0056
Fracture-free life year^c^	0.7995	0.7894	0.0101	0.0064
ICER				
€ per life year gained	233,646			
€ per fracture-free life year gained	141,113			

*OGCM* Orthogeriatric co-management, *SE* Robust standard error

^*^p < .05; ^**^p < .001

^a^Estimated with a gamma regression

^b^Estimated with a two-part model with logistic and gamma parts

^c^Estimated using a linear regression

Both sensitivity analyses (supplementary Tables 3–6; supplementary Fig. 3–4, Additional File 1, appendix D-E, p. 7–13) produced similar results as the main analysis. Notable differences were slightly higher life years and fracture-free life years in the OGCM than in the non-OGCM group of the humerus fracture cohort when accounting for potential hospital clusters and a significantly longer LOS in a rehabilitation facility in the OGCM group of the forearm fracture cohort in the analysis without using hospital volume in the entropy balancing.

## Discussion

Using health and long-term care insurance claims data of patients with forearm or humerus fragility fractures treated in a hospital that was either able to offer OGCM or in a hospital that was not, we analyzed costs in different health sectors and cost-effectiveness in a 1-year follow-up. In both cohorts, the index stay, inpatient, and total costs in an OGCM hospital were higher. We did not find differences concerning life years or fracture-free life years for both cohorts. The probability that treatment in an OGCM hospital was cost-effective exceeded 95% in none of the analyses.

In agreement with a German investigation of hip fractures using insurance claims data [26], we found higher total and inpatient costs after treatment in OGCM hospitals compared to non-OGCM hospitals. As in the current investigation, this was mostly driven by higher index costs. Although this is not in line with two systematic reviews on orthogeriatric care for hip fractures that found lower costs in the orthogeriatric care than the control groups [23, 24], none of the reviewed studies was from Germany. In contrast to the reviewed articles, the LOS was longer, not shorter, in the OGCM groups of the current and other German investigations [22, 26, 27], highlighting difficulties in comparing the LOS across healthcare systems [18] and fracture locations. Thus, the differences in index stay costs were likely driven by a longer LOS in the OGCM group. Providing in-hospital rehabilitation during German OGCM might explain a longer LOS [22]. Accordingly, another German claims data study on hip fractures found that patients from their non-OGCM group were more likely to receive a subsequent rehabilitative treatment than those from their OGCM group [22]. While we did not obtain a longer stay in subsequent rehabilitation facilities in the non-OGCM than in the OGCM groups, fractures of the upper extremities might not result in a stay in a rehabilitation facility (for 1.8% in the forearm and 9% in the humerus fracture cohort, a subsequent rehabilitation stay was recorded). Lastly, the German reimbursement system might encourage a longer LOS to exceed the 14-day threshold as this affects strongly the reimbursement rate [34]. Regarding cost-sectors not affected by the index stay, the only significant difference, higher outpatient hospital costs in the humerus fracture cohort, could not be seen in the sensitivity analyses.

We did not find differences regarding life years in either fracture cohort (only slight benefits in the OGCM group of the humerus fracture cohort in one of the sensitivity analyses). Our results diverge from studies that found decreased mortality or higher life years after orthogeriatric collaboration for hip fractures [22, 23, 26] but are in line with studies on non-hip fractures that did not find a significantly reduced mortality [28–30]. Mortality after forearm and humerus fractures, however, has been shown to be lower than that of other fragility fractures [11, 14, 15]. Hence, the impact of fracture treatment on subsequent mortality is limited. Additionally, as the mortality was shown to be highest directly after the fracture [8], a 1-year follow-up might not have been the optimal investigation period. Concerning fracture-free life years, we did not find differences in either fracture cohort (only slight benefits in the OGCM group of the humerus fracture cohort in one of the sensitivity analyses). However, we might have underestimated differences because, like mortality, refracture risk has been shown to be highest shortly after the initial fracture [42] and we did not consider refractures of the same fracture location in the first 6 weeks after the incident fracture. Overall, in none of the analyses, the probability that treatment in OGCM hospitals compared to non-OGCM hospitals was cost-effective exceeded 95%. While these results are less promising than prior analyses on hip fractures [23, 24, 26], a few limitations apply.

First, our effectiveness outcomes did not reflect the manifold health effects of forearm and humerus fractures. While we included fracture-free life years because upper extremities fractures do not affect the mortality as much as other fracture locations [11, 14, 15] and to account for the increased refracture risk [16], this combined measure is difficult to interpret. Second, we only investigated costs from a payer perspective and lacked a societal view (e.g., lacking costs of informal care). However, the payer perspective likely reflects most of the relevant costs, considering the broad coverage of health services by German statutory health insurance and that direct costs outweigh the indirect costs of fragility fractures [43]. Third, OGCM is an inpatient treatment but some fractures, fractures of the forearm, in particular, are often treated in an outpatient setting [44]. Focusing on inpatient treatments, we likely predominantly selected severe cases, also indicated by high rates of surgical treatments. Moreover, the proportion of surgically treated forearm and humerus fractures in Germany likely is higher than in many other countries. We acknowledge there is no published evidence for any superiority on long term outcomes comparing conservative and operative treatment for wrist [45] or proximal humerus fractures [46]. Fourth, we had to assign patients to study groups at hospital-level. OPS8-550 was recorded for a minority of the cases, indicating that many patients in the OGCM group might not have received OGCM. However, when patients are discharged or die after less than 7 days of treatment, it is impossible to use OPS8-550 even though patients might have benefitted from the multidisciplinary team in an OGCM hospital. Moreover, we assumed that patients treated in hospitals with OGCM benefitted from the local expertise irrespective of whether they received a complex early geriatric rehabilitation (OPS8-550). But the contrary may be true. Resources may have been focused on patients getting a complex early geriatric rehabilitation and other patients may have been neglected.

Last, not all risk factors could be accessed comprehensively: For example, we addressed prior fragility fractures [9] by applying a washout period of 180 days but the refracture risk might be increased for years after an initial fracture [42] exceeding our time horizon. Another important risk factor is osteoporosis and its medication [9]. While balancing for medication-based comorbidities [38] included balancing for osteoporosis medication (including bisphosphonates and combinations with calcium or vitamin D), we could not access baseline differences in bone mineral density – a pivotal risk factor for fragility fractures [9]. Moreover, the detailed analysis of refractures was beyond the scope of this study although forearm and humerus fractures are a risk factor for subsequent fractures [16] and were frequent in our forearm fracture cohort (10.7% of the cases in the non-OGCM, 10.2% in the OGCM group obtained a subsequent fractures) and humerus fracture cohort (11.2% non-OGCM group, 11% OGCM group).

To our knowledge, this was the first study to investigate the costs and cost-effectiveness of OGCM hospitals for fractures of the upper extremities. We used a rich data set of more than 30,000 persons per cohort from the AOK, which covers about one-third of the German population, indicating a rather representative sample for Germany. Moreover, using entropy balancing this comprehensive data set allowed us to achieve almost exact balance (regarding the mean, variance, and skewness) on a wide range of covariates, including age, sex, baseline costs, and medication-based comorbidities to account for the lack of randomization.

## Conclusion

We investigated the costs and cost-effectiveness of treatment of forearm or humerus fracture patients in OGCM hospitals compared to non-OGCM hospitals. Although we did not find the treatment in OGCM hospitals to be cost-effective, future studies are needed. In particular, they should utilize a holistic effectiveness outcome to capture the manifold health effects of orthogeriatric care.

## Supplementary Information

Supplementary Material 1.

## Data Availability

The datasets supporting the conclusions of this article are owned by the German statutory health insurance AOK. Since public deposition of the data would breach ethical and legal compliance, data are only available upon formal request from the research institute of the AOK (WIdO). To request the data please contact the institutional body of the WIdO (wido@wido.bv.aok.de). To fulfill the legal requirements to obtain that kind of data, researchers must obtain permission for a specific research question from the German Federal (Social) Insurance Office. Additionally, researchers must conclude a contract with the statutory health insurance regarding data access which can be requested from the "AOK-Bundesverband GbR" (Federal Association of Local Health Insurance Funds) under http://aok-bv.De/kontakt/. The licensee is permitted to use the data for the purpose of the research proposal within their company, exclusively. Thereby, a company is defined as an economic unit. Licensees are not allowed to pass the data to a third party, or to create software or databases except for scientific publications. Moreover, the study has to be approved by the data protection officer both at the statutory health insurance and the research institute.

## Abbreviations

CEAC: Cost-effectiveness acceptability curve
ICER: Incremental cost-effectiveness ratio
LOS: Length of stay
OGCM: Orthogeriatric co-management
WTP: Willingness-to-pay
