# Correction: Health-economic evaluation of orthogeriatric co-management for patients with forearm or humerus fractures: an analysis of insurance claims data from Germany

**DOI:** 10.1186/s12913-024-11864-6

**Published:** 2024-11-01

**Authors:** Espen Henken, Hans‑Helmut Konig, Clemens Becker, Gisela Buchele, Thomas Friess, Andrea Jaensch, Kilian Rapp, Dietrich Rothenbacher, Claudia Konnopka

**Affiliations:** 1 Department of Health Economics and Health Services Research, University Medical Center Hamburg-Eppendorf, Martinistr. 52, Hamburg, 20246 Germany; 2 Department of Clinical Gerontology, Robert-Bosch-Hospital, Stuttgart, Germany; 3 Institute of Epidemiology and Medical Biometry, Ulm University, Ulm, Germany; 4 AUC - Akademie Der Unfallchirurgie GmbH, Munich, Germany

**Correction to: BMC Health Serv Res 24, 820 (2024)**

**https://doi.org/10.1186/s12913-024-11297-1**

In this article, the authors noted that the data depicted in Supplementary Fig. 1 originally included in Supplementary Material 1 is wrong.

Both the incorrect and corrected version of Supplementary Material 1 is included in this Correction and Supplementary Material 1 has been replaced by the corrected version in the original article.

## Supplementary Information

Supplementary Material 1. Corrected version.

Supplementary Material 2. Incorrect version

