# Supplementary material for: Health-economic evaluation of orthogeriatric co-management for patients with forearm or humerus fractures: an analysis of insurance claims data from Germany
Source: BMC Health Serv Res. 2024 Jul 16;24:820. doi: 10.1186/s12913-024-11297-1 (PMC11253488; doi:10.1186/s12913-024-11297-1)
Supplement: Supplementary file 1 — Supplementary Material 1. [file 12913_2024_11297_MOESM1_ESM.docx]

**Health-economic evaluation of orthogeriatric co-management for patients with forearm or humerus fractures: An analysis of insurance claims data in Germany**

**Supplementary Material**

**Table of content**

p. 1 **Appendix A: Supplementary Information** – **Methods**

p. 2 **Appendix B: Hospital volume**

p. 3-6 **Appendix C: Descriptive statistics for both cohorts**

p. 7-10 **Appendix D: Sensitivity Analysis** – **Accounting for clusters**

p. 11-13 **Appendix E: Sensitivity analysis** – **Not using hospital volume in entropy balancing**

**Appendix A: Supplementary Information** – **Methods**

**Calculation of long-term care costs**

We calculated long-term care costs indirectly as we obtained information on long-term care only on a monthly basis. Thus, we estimated the months in each care level during the baseline and follow-up period and whether patients lived in a nursing home in each respective month as this influences the reimbursement rate. Then, we subtracted the proportion of days per month in which patients had an inpatient hospital stay that lasted longer than 28 days as no long-term care reimbursement is paid in these cases. In Germany, there is a fixed monthly reimbursed rate per care level depending on whether care is applied ambulatory or in a nursing home and on whether it is paid as benefits-in-kind by a professional service or if care is administered informally for example by relatives. We obtained these rates from the Federal Ministry of Health’s website [1]. As we did not know who administered the care services, we used the average of both of the ambulatory rates and multiplied them with the time in each care level.

**Hospital fracture volume**

For the hospital volume, we counted the number of fractures for both fracture types during the study period of 2013-2019 separately for each federal state as indicated by the place of residency of the patients. Then, we weighted the state-specific values by the AOK’s coverage in the respective state which we determined by dividing the number of persons insured with the AOK [2] by the states’ population [3]. We summed these state-specific values to obtain the hospital volume of each hospital.

**Operation and procedure codes indicating surgical treatment**

We used the following operation and procedure codes to indicate a surgical treatment in forearm fractures: 5-79 and 4-9 as the sixth digit, e.g., 5-79009 or 5-79108 as well as 5-824 with 3 or 4 as a fifth digit, e.g., 5-8243 and 5-82440. We used the following operation and procedure codes to indicate a surgical treatment in humerus fractures: 5-79 and 0-3 as the sixth digit, e.g., 5-79000 or 5-79103 as well as 5-824 with 0, 2, 3 or 4 as a fifth digit, e.g., 5-82401.

**Appendix B: Hospital volume**

**Supplementary figure 1** Hospital volume (fracture cases per hospital) for OGCM and non-OGCM hospitals


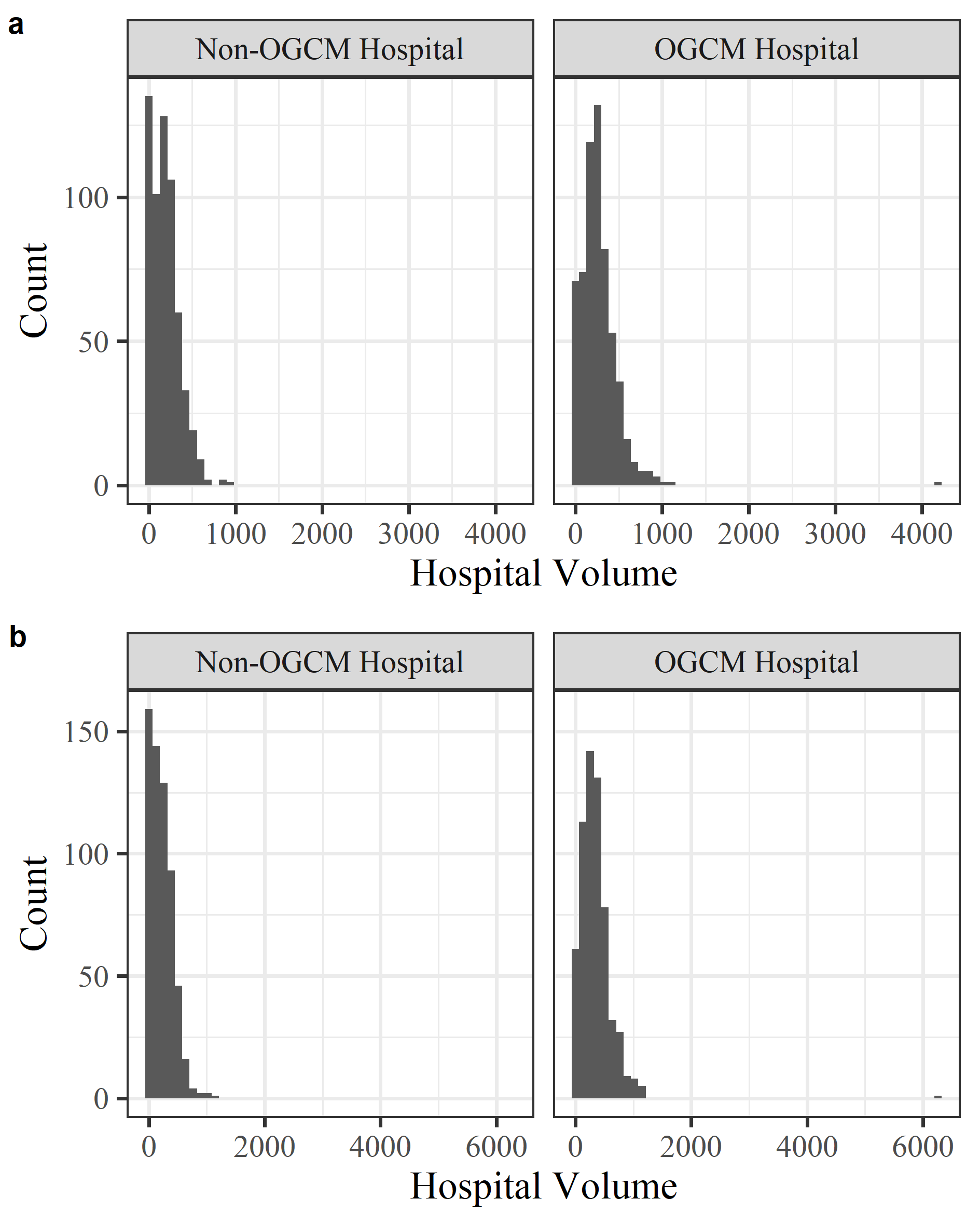


These values were weighted by the insurer’s coverage in the respective federal state. A: Forearm fracture cohort, B: Humeral fracture cohort. All cases belonging to the outlier in the OGCM hospital group were excluded during data preprocessing.

**Appendix C: Descriptive statistics for both cohorts**

| **Supplementary table 1** Descriptive statistics before and after entropy balancing for forearm fractures | | | |
| --- | --- | --- | --- |
| Baseline: 1 year | OGCM group (N=19,900) | Non-OGCM group (N=11,657) | |
|  |  | Before EB | After EB |
| Female sex [%] | 92.74 (25.94) | 92.85 (25.77) | 92.74 (25.94) |
| Age: Mean [years] | 85.84 (4.19) | 85.7 (4.04) | 85.84 (4.19) |
| Forearm fracture cases per hospital: Mean | 416 (210) | 302 (153) | 416 (210) |
| Treatment in 2014 [%] | 17.14 (37.69) | 24.17 (42.82) | 17.18 (37.72) |
| Treatment in 2015 [%] | 19.51 (39.63) | 21.44 (41.04) | 19.51 (39.63) |
| Treatment in 2016 [%] | 19.73 (39.8) | 19.74 (39.8) | 19.73 (39.8) |
| Treatment in 2017 [%] | 21.18 (40.86) | 18.2 (38.59) | 21.17 (40.85) |
| Care dependence during baseline: Mean [months] | | | |
| nursing home | 1.13 (3.34) | 1.1 (3.29) | 1.13 (3.34) |
| care level 1 | 0.04 (0.55) | 0.03 (0.46) | 0.04 (0.55) |
| care level 2 | 2.21 (4.36) | 2.12 (4.26) | 2.21 (4.36) |
| care level 3 | 1.4 (3.58) | 1.28 (3.42) | 1.4 (3.58) |
| care level 4 | 0.68 (2.59) | 0.67 (2.57) | 0.68 (2.59) |
| care level 5 | 0.09 (0.94) | 0.09 (0.98) | 0.09 (0.94) |
| Costs during baseline: Mean [€] | | | |
| for inpatient hospital treatment | 2,624 (5,169) | 2,402 (4,920) | 2,623 (5,168) |
| for medication | 1,006 (1,239) | 971 (1,180) | 1,005 (1,239) |
| for outpatient treatment | 952 (634) | 968 (649) | 953 (634) |
| for outpatient hospital treatment | 24.36 (108) | 23.53 (102) | 24.36 (108) |
| for medical devices | 146 (353) | 146 (344) | 146 (353) |
| for long-term care | 4,001 (5,918) | 3,842 (5,890) | 4,001 (5,918) |
| Medication-based comorbidities [%] | | | |
| Acid related disorders | 44.82 (49.73) | 45.29 (49.78) | 44.82 (49.73) |
| Bone diseases (osteoporosis) | 11.42 (31.81) | 11.86 (32.34) | 11.42 (31.81) |
| Cancer | 0.49 (7) | 0.54 (7.33) | 0.49 (7) |
| Cardiovascular diseases (incl.   hypertension) | 85.74 (34.96) | 85.52 (35.19) | 85.74 (34.96) |
| Dementia | 6.17 (24.06) | 6.02 (23.79) | 6.17 (24.06) |
| Diabetes mellitus | 16.28 (36.92) | 16.2 (36.84) | 16.28 (36.92) |
| Epilepsy | 8.91 (28.5) | 8.22 (27.47) | 8.91 (28.49) |
| Glaucoma | 10.01 (30.01) | 9.9 (29.87) | 10 (30.01) |
| Gout, Hyperuricemia | 9.92 (29.9) | 10.79 (31.03) | 9.93 (29.9) |
| HIV | Excluded due to less than 50 observations | | |
| Hyperlipidemia | 30.89 (46.21) | 30.35 (45.98) | 30.89 (46.21) |
| Intestinal inflammatory diseases | 0.78 (8.82) | 0.88 (9.36) | 0.78 (8.82) |
| Iron deficiency anemia | 4.83 (21.45) | 4.45 (20.63) | 4.83 (21.45) |
| Migraines | Excluded due to less than 50 observations | | |
| Pain | 50.54 (50) | 48.89 (49.99) | 50.54 (50) |
| Parkinson’s disease | 5.83 (23.43) | 5.41 (22.63) | 5.83 (23.43) |
| Psychological disorders (sleep disorder,   depression) | 30.36 (45.98) | 31.72 (46.54) | 30.36 (45.98) |
| Psychoses | 12.41 (32.97) | 11.78 (32.24) | 12.4 (32.96) |
| Respiratory illness (asthma, COPD) | 12.97 (33.6) | 12.7 (33.3) | 12.97 (33.6) |
| Rheumatologic conditions | 33.58 (47.23) | 35.14 (47.74) | 33.59 (47.23) |
| Thyroid disorders | 25.48 (43.58) | 25.22 (43.43) | 25.48 (43.58) |
| Tuberculosis | Excluded due to less than 50 observations | | |
| Standard deviation is stated in parentheses; EB = Entropy balancing; OGCM = Orthogeriatric co-management | | | |

| **Supplementary table 2** Descriptive statistics before and after entropy balancing for humeral fractures | | | |
| --- | --- | --- | --- |
| Baseline: 1 year | OGCM group (N=24,997) | Non-OGCM group (N=14,096) | |
|  |  | Before EB | After EB |
| Female sex [%] | 85.29 (35.43) | 85.29 (35.43) | 85.29 (35.42) |
| Age: Mean [years] | 86.4 (4.43) | 86.37 (4.4) | 86.4 (4.43) |
| Humerus fracture cases per hospital: Mean | 512 (251) | 350 (165) | 509 (252) |
| Treatment in 2014 [%] | 17.28 (37.81) | 23.32 (42.29) | 17.35 (37.87) |
| Treatment in 2015 [%] | 18.64 (38.95) | 21.16 (40.85) | 18.66 (38.96) |
| Treatment in 2016 [%] | 20.2 (40.15) | 19.25 (39.43) | 20.19 (40.14) |
| Treatment in 2017 [%] | 21.19 (40.86) | 19.4 (39.54) | 21.15 (40.84) |
| Care dependence during baseline: Mean [months] | | | |
| nursing home | 1.61 (3.93) | 1.62 (3.93) | 1.61 (3.93) |
| care level 1 | 0.05 (0.59) | 0.04 (0.54) | 0.05 (0.59) |
| care level 2 | 2.51 (4.55) | 2.47 (4.5) | 2.51 (4.54) |
| care level 3 | 1.82 (3.99) | 1.75 (3.9) | 1.82 (3.99) |
| care level 4 | 1.08 (3.22) | 1.04 (3.15) | 1.08 (3.22) |
| care level 5 | 0.23 (1.56) | 0.21 (1.48) | 0.23 (1.56) |
| Costs during baseline: Mean [€] | | | |
| for inpatient hospital treatment | 3,062 (5,762) | 2,779 (5,493) | 3,061 (5,761) |
| for medication | 1,144 (1,381) | 1,098 (1,319) | 1,144 (1,380) |
| for outpatient treatment | 981 (708) | 993 (706) | 981 (708) |
| for outpatient hospital treatment | 24.44 (116) | 23.27 (109) | 24.45 (116) |
| for medical devices | 166 (420) | 175 (431) | 166 (420) |
| for long-term care | 5,528 (6,792) | 5,372 (6,767) | 5,527 (6,792) |
| Medication-based comorbidities [%] | | | |
| Acid related disorders | 47.02 (49.91) | 47.43 (49.94) | 47.03 (49.91) |
| Bone diseases (osteoporosis) | 8.79 (28.31) | 8.34 (27.65) | 8.78 (28.3) |
| Cancer | 0.46 (6.8) | 0.38 (6.12) | 0.46 (6.8) |
| Cardiovascular diseases (incl.   hypertension) | 86.42 (34.26) | 86.43 (34.25) | 86.43 (34.25) |
| Dementia | 7.09 (25.66) | 6.96 (25.45) | 7.08 (25.65) |
| Diabetes mellitus | 22.45 (41.72) | 21.97 (41.41) | 22.44 (41.72) |
| Epilepsy | 9.3 (29.05) | 9.19 (28.9) | 9.3 (29.04) |
| Glaucoma | 9.67 (29.55) | 9.85 (29.81) | 9.67 (29.55) |
| Gout, Hyperuricemia | 12.29 (32.83) | 12.63 (33.22) | 12.29 (32.83) |
| HIV | Excluded due to less than 50 observations | | |
| Hyperlipidemia | 30.74 (46.14) | 29.81 (45.74) | 30.74 (46.14) |
| Intestinal inflammatory diseases | 0.84 (9.11) | 0.84 (9.11) | 0.84 (9.11) |
| Iron deficiency anemia | 5.99 (23.74) | 5.47 (22.74) | 5.99 (23.73) |
| Migraines | Excluded due to less than 50 observations | | |
| Pain | 52.47 (49.94) | 51.28 (49.99) | 52.47 (49.94) |
| Parkinson’s disease | 6.12 (23.98) | 6.07 (23.87) | 6.12 (23.98) |
| Psychological disorders (sleep disorder,   depression) | 31.97 (46.64) | 32.21 (46.73) | 31.98 (46.64) |
| Psychoses | 15.76 (36.44) | 15.44 (36.14) | 15.75 (36.43) |
| Respiratory illness (asthma, COPD) | 13.2 (33.85) | 12.51 (33.08) | 13.2 (33.85) |
| Rheumatologic conditions | 30.12 (45.88) | 31.9 (46.61) | 30.15 (45.89) |
| Thyroid disorders | 24.09 (42.76) | 24.93 (43.26) | 24.1 (42.77) |
| Tuberculosis | Excluded due to less than 50 observations | | |
| Standard deviation is stated in parentheses; EB = Entropy balancing; OGCM = Orthogeriatric co-management | | | |

**Appendix D: Sensitivity Analysis** – **Accounting for clusters**

We recalculated all analyses including a random intercept term for hospitals to account for potential hospital clusters. Two-part models with random intercept terms in both parts of the model did not converge. Therefore, we only used a random intercept term in the first (logistic) part of the two-part models because there it affects all cases not only those with non-zero values. The incremental cost-effectiveness ratios were calculated using the marginal effects of these models. We also recalculated the cost-effectiveness acceptability curves with a random intercept term for hospitals using STATA 16 (StataCorp, College Station, USA). Estimated costs and outcomes can be found in supplementary Tables 3 and 4 and the cost-effectiveness acceptability curves can be found in supplementary Figure 2.

| **Supplementary table 3** Costs and outcome estimates for forearm fractures estimated with random intercepts for hospitals | | | | |
| --- | --- | --- | --- | --- |
| Outcome | OGCM group  (n = 19,900) | Non-OGCM group  (n = 11,657) | Difference | SE |
| Costs [€] |  |  |  |  |
| Total^a^ | 16,879 | 16,110 | 769*** | 179 |
| Inpatient^a^ | 8,281 | 7,622 | 659*** | 135 |
| Thereof during index stay^a^ | 4,172 | 3,570 | 601*** | 59.09 |
| Medication^b^ | 1,087 | 1,087 | 0.1405 | 23.05 |
| Outpatient^b^ | 1,000 | 1,016 | -15.86 | 17.55 |
| Outpatient hospital^b^ | 29.87 | 31.63 | -1.75 | 2.25 |
| Medical devices^b^ | 315 | 312 | 2.74 | 7.78 |
| Long-term care^c^ | 5,875 | 5,828 | 46.87 | 100 |
| Length of stay [days] |  |  |  |  |
| Total stay^a^ | 7.82 | 6.4 | 1.41*** | 0.1764 |
| Thereof in hospital^a^ | 7.4 | 6.1 | 1.3*** | 0.1498 |
| Thereof in rehabilitation facility^b^ | 0.1959 | 0.1638 | 0.0321 | 0.0247 |
| Effectiveness |  |  |  |  |
| Life year^c^ | 0.9302 | 0.9315 | -0.0013 | 0.0027 |
| Fracture-free life year^c^ | 0.8693 | 0.8654 | 0.0039 | 0.0043 |
| ICER |  |  |  |  |
| € per life year gained | Domintaed^d^ |  |  |  |
| € per fracture-free life year gained | 197,246 |  |  |  |
| * *p* < .05; ** *p* < .01; *** *p* < .001 ^a^ estimated with a gamma regression with random intercept term for hospitals; ^b^ estimated with a two-part model with logistic and gamma parts with random intercept term for hospitals in logistic part; ^c^ estimated with a linear mixed regression with random intercept term for hospitals; ^d^ OGCM was more costly and less effective than non-OGCM group; OGCM = Orthogeriatric co-management; OGCM = Orthogeriatric co-management; SE = Robust standard error | | | | |

| **Supplementary table 4** Costs and outcome estimates for humeral fractures estimated with random intercepts for hospitals | | | | |
| --- | --- | --- | --- | --- |
| Outcome | OGCM group  (n = 24,997) | Non-OGCM group  (n = 14,096) | Difference | SE |
| Costs [€] |  |  |  |  |
| Total^a^ | 22,008 | 21,021 | 987*** | 189 |
| Inpatient^a^ | 11,627 | 10,588 | 1,038*** | 165 |
| Thereof during index stay^a^ | 6,682 | 5,610 | 1,072*** | 100 |
| Medication^b^ | 1,199 | 1,162 | 37.38 | 48.67 |
| Outpatient^b^ | 966 | 964 | 1.83 | 23.07 |
| Outpatient hospital^b^ | 21.54 | 21.18 | 0.3573 | 2.2 |
| Medical devices^b^ | 446 | 462 | -15.47 | 7.93 |
| Long-term care^c^ | 7,390 | 7,418 | -28.15 | 92.53 |
| Length of stay [days] |  |  |  |  |
| Total stay^a^ | 15.38 | 12.53 | 2.85*** | 0.3186 |
| Thereof in hospital^a^ | 13.35 | 10.81 | 2.54*** | 0.2721 |
| Thereof in rehabilitation facility^b^ | 1.23 | 1.11 | 0.1245 | 0.0991 |
| Effectiveness |  |  |  |  |
| Life year^c^ | 0.8534 | 0.846 | 0.0074* | 0.0036 |
| Fracture-free life year^c^ | 0.7996 | 0.7898 | 0.0098* | 0.0044 |
| ICER |  |  |  |  |
| € per life year gained | 133,359 |  |  |  |
| € per fracture-free life year gained | 100,700 |  |  |  |
| * *p* < .05; ** *p* < .01; *** *p* < .001 ^a^ estimated with a gamma regression with random intercept term for hospitals; ^b^ estimated with a two-part model with logistic and gamma parts with random intercept term for hospitals in logistic part; ^c^ estimated with a linear mixed regression with random intercept term for hospitals; OGCM = Orthogeriatric co-management; SE = Robust standard error | | | | |

**Supplementary figure 3** Cost-effectiveness acceptability curves for total costs per (fracture-free) life year gained for estimated with random intercepts for hospitals


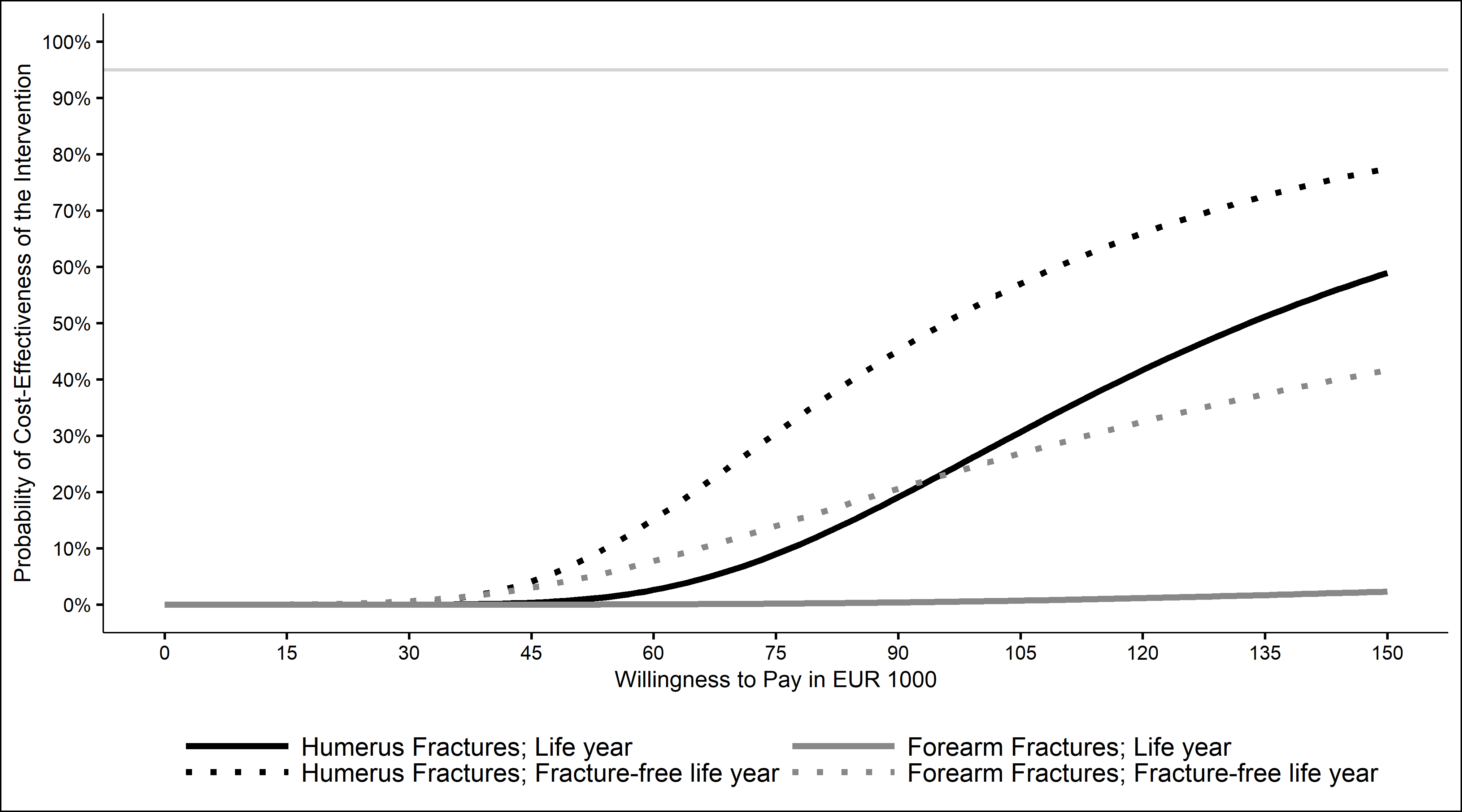


**Appendix E: Sensitivity analysis** – **Not using hospital volume in entropy balancing**

| **Supplementary table 5** Costs and outcome estimates for forearm fractures without using hospital volume in the enropy balancing | | | | |
| --- | --- | --- | --- | --- |
| Outcome | OGCM group  (n = 19,900) | Non-OGCM group  (n = 11,657) | Difference | SE |
| Costs [€] |  |  |  |  |
| Total^a^ | 16,855 | 16,134 | 721*** | 138 |
| Inpatient^a^ | 8,326 | 7,591 | 734*** | 85.88 |
| Thereof during index stay^a^ | 4,201 | 3,641 | 560*** | 26.72 |
| Medication^b^ | 1,084 | 1,096 | -12.06 | 15.65 |
| Outpatient^b^ | 997 | 1,019 | -21.93** | 8.01 |
| Outpatient hospital^b^ | 34.06 | 36.59 | -2.54 | 1.56 |
| Medical devices^b^ | 314 | 317 | -2.6 | 5.31 |
| Long-term care^c^ | 5,849 | 5,847 | 2.06 | 79.18 |
| Length of stay [days] |  |  |  |  |
| Total stay^a^ | 8.21 | 6.44 | 1.77*** | 0.0948 |
| Thereof in hospital^a^ | 7.79 | 6.1 | 1.69*** | 0.0819 |
| Thereof in rehabilitation facility^b^ | 0.4167 | 0.3415 | 0.0752* | 0.0357 |
| Effectiveness^c^ |  |  |  |  |
| Life year^c^ | 0.93 | 0.9316 | -0.0016 | 0.0026 |
| Fracture-free life year^c^ | 0.8696 | 0.8666 | 0.003 | 0.0034 |
| ICER^c^ |  |  |  |  |
| ICER life year^c^ | Dominated^d^ |  |  |  |
| ICER fracture-free life year^c^ | 240,385 |  |  |  |
| * *p* < .05; ** *p* < .01; *** *p* < .001 ^a^ estimated with a gamma regression; ^b^ estimated with a two-part model with logistic and gamma parts; ^c^ estimated with a linear regression; ^d^ OGCM was more costly and less effective than non-OGCM group; OGCM = Orthogeriatric co-management; SE = Robust standard error | | | | |

| **Supplementary table 6** Costs and outcome estimates for humerus fractures without using hospital volume in the enropy balancing | | | | |
| --- | --- | --- | --- | --- |
| Outcome | OGCM group  (n = 24,997) | Non-OGCM group  (n = 14,096) | Difference | SE |
| Costs [€] |  |  |  |  |
| ... Total^a^ | 21,994 | 20,725 | 1270*** | 136 |
| ... Inpatient^a^ | 11,715 | 10,454 | 1262*** | 93.1 |
| ... Thereof during index stay^a^ | 6,776 | 5,641 | 1135*** | 42.39 |
| ... Medication^b^ | 1,197 | 1,182 | 15 | 14.74 |
| ... Outpatient^b^ | 964 | 979 | -15.54* | 7.74 |
| ... Outpatient hospital^b^ | 27.7 | 25.6 | 2.11 | 1.26 |
| ... Medical devices^b^ | 445 | 459 | -13.9* | 5.75 |
| ... Long-term care^c^ | 7,377 | 7,345 | 32.8 | 73.98 |
| Length of stay [days] |  |  |  |  |
| ... Total stay^a^ | 15.8 | 12.11 | 3.69*** | 0.1341 |
| ... Thereof in hospital^a^ | 13.79 | 10.18 | 3.61*** | 0.102 |
| ... Thereof in rehabilitation facility^b^ | 2.01 | 1.93 | 0.0823 | 0.071 |
| Effectiveness^c^ |  |  |  |  |
| ... Life year^c^ | 0.8533 | 0.8512 | 0.0021 | 0.0033 |
| ... Fracture-free life year^c^ | 0.7995 | 0.7964 | 0.003 | 0.0037 |
| ICER^c^ |  |  |  |  |
| ICER life year^c^ | 604,669 |  |  |  |
| ICER fracture-free life year^c^ | 423,268 |  |  |  |
| * *p* < .05; ** *p* < .01; *** *p* < .001 ^a^ estimated with a gamma regression; ^b^ estimated with a two-part model with logistic and gamma parts; ^c^ estimated with a linear regression; OGCM = Orthogeriatric co-management; SE = Robust standard error | | | | |

**Supplementary figure 4** Cost-effectiveness acceptability curves for total costs per (fracture-free) life year gained without using hospital volume in the enropy balancing


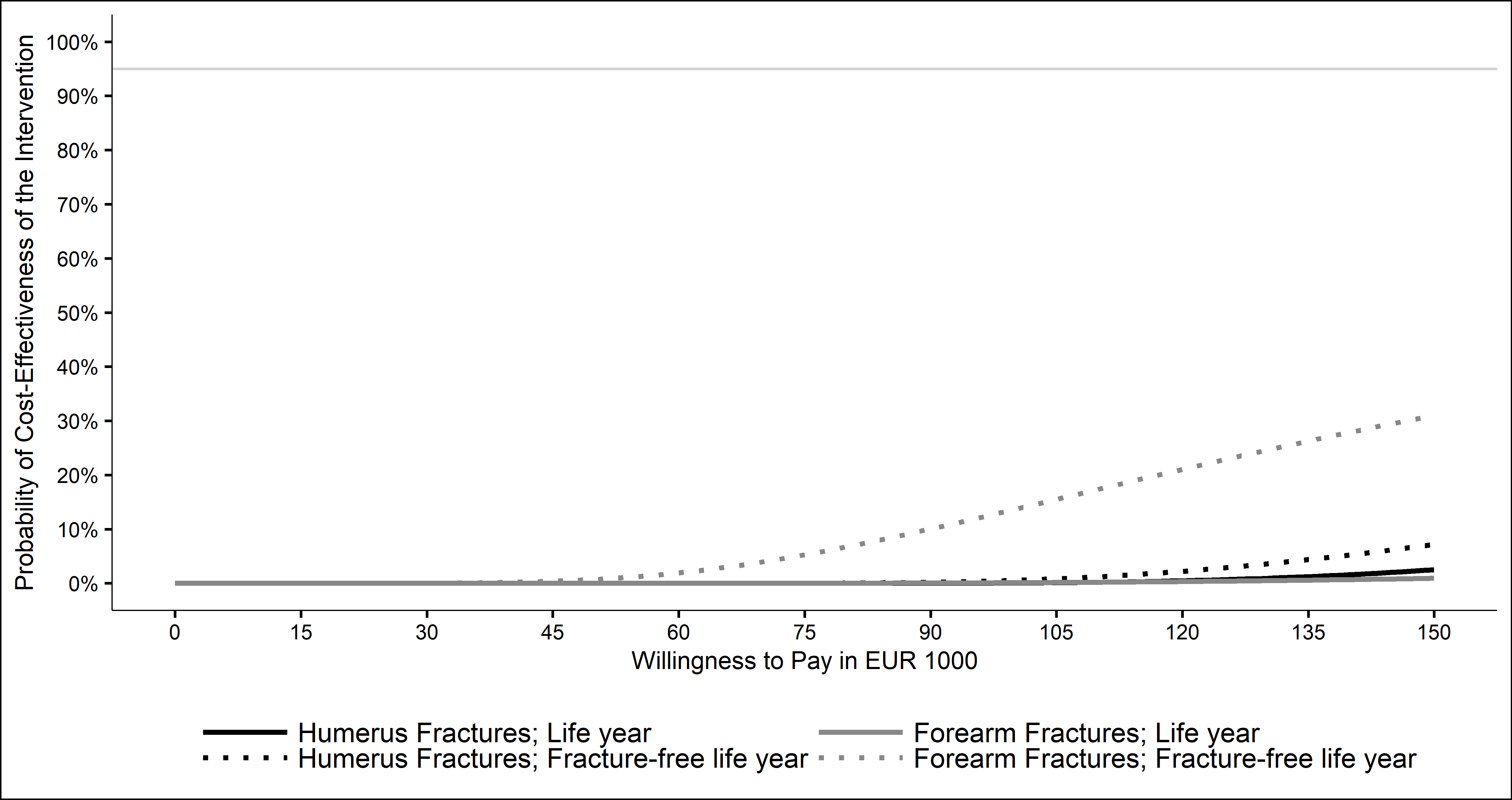


**References**

1. Bundesministerium für Gesundheit. *Ratgeber Pflege: Alles, was Sie zum Thema Pflege wissen sollten*. <https://www.bundesgesundheitsministerium.de/fileadmin/Dateien/5_Publikationen/Pflege/Broschueren/Ratgeber_Pflege_Okt_2019__barrPDF.pdf> (accessed 09 December 2021)

2. Bundesministerium für Gesundheit. *Mitglieder und Versicherte der Gesetzlichen Krankenversicherung (GKV)*. <https://www.bundesgesundheitsministerium.de/themen/krankenversicherung/zahlen-und-fakten-zur-krankenversicherung/mitglieder-und-versicherte.html> (accessed 01 July 2022)

3. Statistisches Bundesamt. *Fortschreibung des Bevölkerungsstandes*. <https://www-genesis.destatis.de/genesis//online?operation=table&code=12411-0010&bypass=true&levelindex=0&levelid=1664879467171#abreadcrumb> (accessed 01 July 2022)
